# Synthesis and Thermoelectric Properties in the 2D Ti_1 – *x*_Nb*_x_*S_3_ Trichalcogenides

**DOI:** 10.3390/ma8052514

**Published:** 2015-05-11

**Authors:** Patrick R. N. Misse, David Berthebaud, Oleg I. Lebedev, Antoine Maignan, Emmanuel Guilmeau

**Affiliations:** Laboratoire CRISMAT, UMR 6508 CNRS/ENSICAEN, 6 bd Maréchal Juin, Caen Cedex 4 14050, France; E-Mails: patrick.misse-ndong@ensicaen.fr (P.R.N.M.); david.berthebaud@ensicaen.fr (D.B.); oleg.lebedev@ensicaen.fr (O.I.L.); antoine.maignan@ensicaen.fr (A.M.)

**Keywords:** sulfur, transition metal sulfides, niobium, titanium trisulfide, thermoelectric, electrical properties, thermal conductivity, Seebeck coefficient

## Abstract

A solid solution of Ti_1 − *x*_Nb*_x_*S_3_ composition (*x* = 0, 0.05, 0.07, 0.10) was synthesized by solid-liquid-vapor reaction followed by spark plasma sintering. The obtained compounds crystallize in the monoclinic ZrSe_3_ structure type. For the *x* = 0.07 sample, a mixture of both A and B variants of the *MX_3_* structure is evidenced by transmission electron microscopy. This result contrasts with those of pristine TiS_3_, prepared within the same conditions, which crystallizes as a large majority of A variant. Thermoelectric properties were investigated in the temperature range 323 to 523 K. A decrease in the electrical resistivity and absolute value of the Seebeck coefficient is observed when increasing *x* due to electron doping. The lattice component of the thermal conductivity is effectively reduced by the Nb for Ti substitution through a mass fluctuation effect and/or a disorder effect created by the mixture of both A and B variants. Due to the low carrier concentration and the semiconductor character of the doped compounds, the too low power factor values leads to *ZT* values that remain smaller by a factor of 50 than those of the TiS_2_ layered compound.

## 1. Introduction

Thermoelectric materials have attracted much attention in recent years for possible applications as environmentally electric-power generators [1]. To qualify the thermoelectric performance of a material, the dimensionless thermoelectric figure of merit, *ZT = S^2^T/*ρκ, is used where *S* is the Seebeck coefficient, ρ is the electrical resistivity, κ is the thermal conductivity, and *T* the absolute temperature. Currently, the best performances for low and medium temperatures range belong to Bi_2_Te_3_ intermetallics with optimum *ZT* values around 1 at 400 K. However, tellurium is toxic, scarce and expensive and this prevents the use of Bi_2_Te_3_ bulk thermoelectric materials for large scale applications. Thus, one of the current main interests in research on thermoelectric materials is to develop new materials with higher efficiency for room and medium temperature range (*i.e.*, below 400 °C).

**Figure 1:**
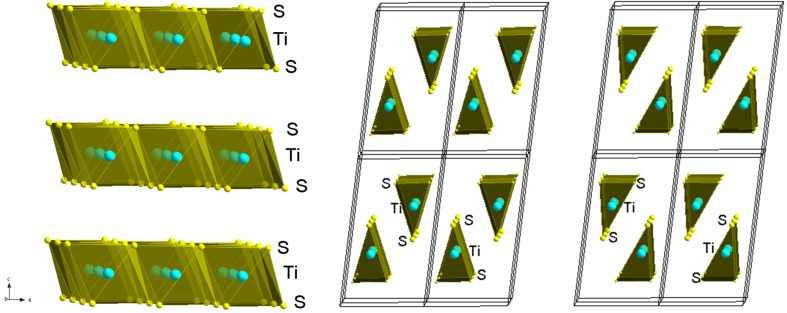
Structure views of TiS_2_, and the A- and B-variants of TiS_3_ (From left to right).

Ten years ago, Imai *et al.* reported a large value for the thermopower in the CdI_2_-type TiS_2_ sulfide (*S* = −250 μV/K at 300 K; n-type behavior) (Figure 1) and relatively low and metallic-like resistivity (ρ = 1.7 mΩ·cm at 300 K) [2]. However, no great efforts have been devoted to this compound in the following years and, only recently, several studies have shown the great potential of this compound for low and medium temperature applications [3,4,5,6,7]. At 300 K, power factor values (*PF = S^2^/*ρ) up to 1.7 mW/mK^2^ similar to those of Bi_2_Te_3_ have been obtained in dense bulk TiS_2_ based compounds [4,8]. Nevertheless, because of its large lattice thermal conductivity, maximum *ZT* values in TiS_2_ are equal to 0.15 at *RT* and around 0.5 at 700 K, *i.e.*, much smaller than in Bi_2_Te_3_. Accordingly, the reduction of the thermal conductivity in TiS_2_ is needed for improving the thermoelectric efficiency for practical applications at room or medium temperature.

With the aim to find lower thermal conductivity in other transition metal sulfides of low structural dimensionality, we have recently studied the thermoelectric properties of the TiS_3_ trichalcogenide [9]. Like other transition metal *MX*_3_ trichalcogenides (*M* = Zr, or Hf, and *X* = S, Se, Te), the TiS_3_ ceramic tends to be made of crystallites exhibiting shapes of fibrous ribbons despite the 2D feature of the structure coming from the S bilayers bonded by van der Waals forces and stacked along the *c* direction (Figure 1) [10,11]. In the structure, each metal *M* is surrounded by six *X* atoms to form a trigonal prism. Depending on the [*M*, *X*] couple, two very close structures exist, noted A (ZrSe_3_-type) and B (TiS_3_-type), belonging both to the P2_1_/*m* monoclinic space group (Figure 1) with similar parameters (*a* ≈ 5 Å, *b* ≈ 3.5 Å, and *c* ≈ 9 Å with β ≈ 97.5°). In a recent investigation [9], it was shown that TiS_3_ can be stabilized in the A-variant type structure by spark plasma sintering (SPS) densification. In this A variant, the Ti cations form chains along the *b* axis against ladders along the same direction in the B variant. In addition, the results of the investigation revealed that, despite moderately low thermal conductivity values and a large absolute value of its Seebeck coefficient at high temperature, the TiS_3_ dense ceramic exhibits too low power factor values to be of significant interest for thermoelectric applications. When compared to TiS_2_, the electrical resistivity of TiS_3_ is found 3 orders of magnitude higher which is mainly related to its lower charge carrier concentration (~10^18^ cm^−3^) and larger band gap (0.9 eV). Thus, electron doping by chemical substitution/intercalation is needed for improving the electrical performances. For that purpose, one of the obvious approaches is to substitute *d*^1^ cations for Ti^4+^ (*d*^0^), *i.e.*, 4*d*^1^ (Nb^4+^) or 5*d*^1^ (Ta^4+^). This has been successful to create electrons in TiS_2_ [12,13]. A second beneficial effect expected from such substitutions, first proposed by Ioffe in 1956 [14], is the fact that the alloying induced by such Ti_1 − *x*_M*_x_*S_3_ substitutions creates point defects scattering responsible for both mass and strain fluctuations which are efficient scatter centers for high frequency phonons. For a solid solution, an improvement in the *ZT* values will be obtained providing the mobility to lattice thermal conductivity ratio is larger than those of the pristine end members constituting the solid solution. Motivated by the effectiveness of the solid solution approach in TiS_2_ to improve *ZT* values [7], we report therein on the synthesis, structure and thermoelectric properties of dense ceramics of the Ti_1 − *x*_Nb*_x_*S_3_ series.

## 2. Results and Discussion

**Figure 2:**
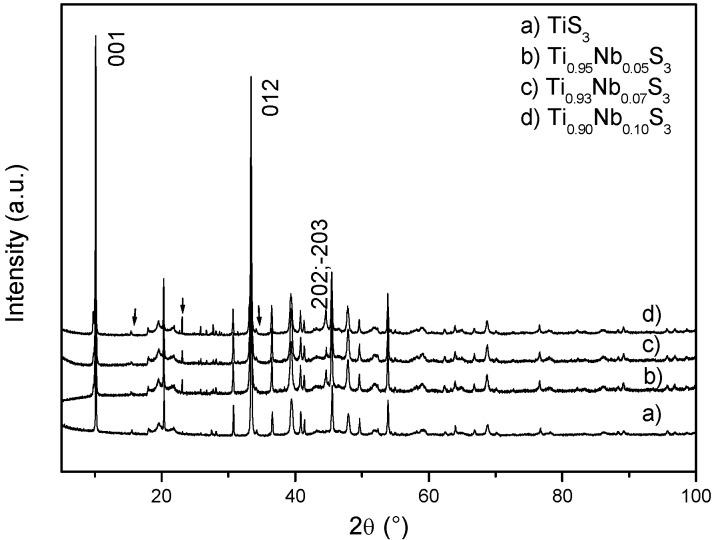
X-ray diffraction (XRD) patterns of Ti_1 − *x*_Nb*_x_*S_3_ (*x* = 0, 0.05, 0.07, 0.10) samples obtained after Spark Plasma Sintering (SPS). Relevant *hkl* index are given. Arrows show extra peaks of unreacted pure sulfur.

After SPS, the purity of the synthesized samples was first analyzed by X-ray powder diffraction (XRD). At room temperature powder, the corresponding patterns of samples with starting composition Ti_1 − *x*_Nb*_x_*S_3_ (*x* = 0.05, 0.07, 0.10), (Figure 2) show that all the compounds crystallize in the ZrSe_3_ structural type (SG: *P*2_1_/*m*) [10]. The XRD pattern of the pure TiS_3_ sample is also shown together with the niobium doped samples. Similar anisotropic disorder and strong broadening of peaks is observed for the niobium doped samples and the TiS_3_ pure sample, as already observed in the previous study of the “SPS” TiS_3_. Though such structural disorders make difficult an accurate le Bail fit or Rietveld refinement to be performed, no significant evolution of the unit cell parameters with *x* was detected. On Figure 1, the extra peaks correspond to traces of pure sulfur. For higher content of niobium (*x* > 0.10), extra peaks corresponding to NbS_3_ were systematically observed in the patterns. Consequently, in the following, these multiphase compounds (*x* > 0.10) were excluded. To go further in the structural analysis, the determination of the TiS_3_ variant nature obtained from XRD analysis was attempted. In the previous study of the pure TiS_3_ densified by SPS, the A-variant was stabilized as the majority phase. One of the signature was the ratio between the (200, −201, and −112) and (201, −202, and 112) sets of peak in the XRD pattern [9]. In the case of Ti_1 − *x*_Nb*_x_*S_3_ (*x* = 0.05, 0.07, 0.10) samples, even if similar ratios of the (200, −201, and −112) and (201, −202, and 112) sets of peak are observed as expected for the A-type, the observed stronger intensity for the (202, −203) set of peaks at around 44.5° would correspond to the calculated theoretical pattern of the B-variant.

**Figure 3:**
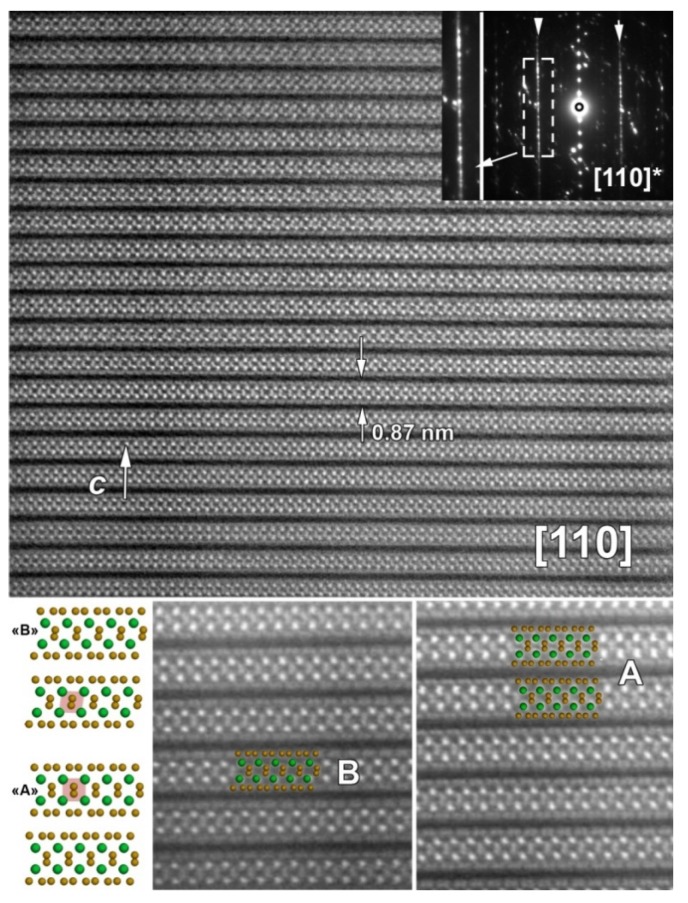
High-resolution (**110**) high angular annular dark field scanning transmission electron microscopy (HAADF-STEM) image of Ti_0.93_Nb_0.07_S_3_ sample and corresponding electron diffraction (ED) pattern. On bottom left panel two possible structure models, **B** and **A** variants are viewed along (**110**) zone axis (Ti/Nb—Green, S—Yellow). Bottom center and right panels show enlargement HAADF-STEM image together with overlays **B** and **A** variants respectively, evidencing the mixture of both variants at nanoscale within single crystallite.

High angular annular dark field scanning transmission electron microscopy (HAADF-STEM) study was undertaken to discriminate between the A and B variants. In principle, the (100) direction allows to distinguish between the variants as well as (110). On Figure 3 (bottom left panel), structure models of A and B variants viewed along (110) zone axis are shown. The A-variant structure exhibits quite perfect square like arrangements of Ti atoms and straight vertical dumbbells of S atoms in between when for the B-variant, the Ti squares are slightly distorted and dumbbells are tilted from vertical position respectively. In enlarged HAADF-STEM images (bottom center and right panels), the structural models of the A and B variants are overlaid with different areas of the sample. A and B variants are found to match perfectly on different areas with the acquired images, supporting the preliminary XRD analysis which pointed out the mixture of both variants A and B in the sample. Those observations are supported by the corresponding electron diffraction (ED) pattern where the diffuse streak lines indicate disorder along the *c*-axis (enlargement of ED pattern on Figure 3) [15].

This structural part points towards the fact that only 7% of Nb for Ti substitution favors a mixture of A and B variants, contrasting with the majority nature of the A variant in the pristine TiS_3_ prepared in the same conditions.

**Figure 4:**
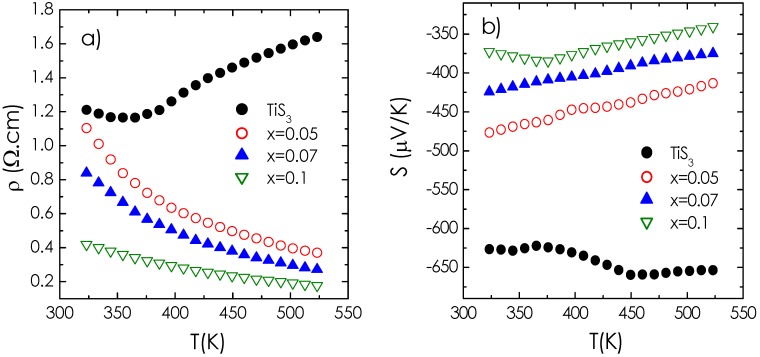
Temperature dependence of (**a**) electrical resistivity and (**b**) Seebeck coefficient in the Ti_1 − *x*_Nb*_x_*S_3_ series.

The temperature dependence of the electrical resistivity in the Ti_1 − *x*_Nb*_x_*S_3_ series is displayed in Figure 4a. The measurements were limited to a maximum operating temperature of 523 K due to the possible decomposition of the phase above this temperature. Finkman and Fisher [16] found that, above 400 K, the results of their investigation on TiS_3_ became irreproducible. This was also suggested recently [9] as a divergence of the S(T) curve above T ~ 600 K was observed, which could indicate a decomposition of TiS_3_ in TiS_2_.

For TiS_3_, as *T* increases, a typical semiconductor to metal transition is observed around 350 K with a minimum resistivity value of ρ = 1.2 Ω·cm at this temperature. The latter is very similar to the one reported on single crystals, with ρ = 3 Ω·cm at 300 K [17]. At low temperature, a semiconductor behavior was reported in TiS_3_ [18] and in NbS_3_ [19,20]. Endo *et al.* have shown that for both phases, the Fermi level lies above the top of valence band, meaning that the compounds are n-type semiconductors [21,22]. In TiS_3_, the band gap is found around 0.9 eV [18,23] with a large Seebeck coefficient of about *S* = −636 µV/K at 300 K [17]. Our data are in agreement with this (Figure 4b). This reflects the empty character of the 3*d*^0^ orbitals for Ti^4+^ according to the Ti^4+^S^2−^ (S_2_)^2−^ formal formula. As soon as 5% at. -Nb is substituted for Ti, a change to a much more conducting behavior is evidenced with a dramatic drop of ρ at high temperature as *x* increases, and the suppression of the metal-like to insulator transition of TiS_3_. The activation energies calculated for the 325 K < *T* < 525 K region have values 60 meV < *E_A_* < 80 meV which are much smaller than those deduced from the TiS_3_ ρ (*T*) curve in the low temperature regime [18,23]. This points towards a different mechanism for the transport properties with a much smaller direct band gap in the case of Nb-doped TiS_3_ samples. The calculated mobilities with values close to 5 cm^2^V^−1^s^−1^ for all *x* values support the fact that the niobium does not scatter the charge carriers. Nevertheless, the ρ decrease with *x* in Ti_1 − *x*_Nb*_x_*S_3_, already found in the Ti_1 − *x*_*M_x_*S_2_ (*M* = Nb, Ta) series [12,13,24,25], can be explained by the partial filling of the conduction band, when Ti^4+^ (*d^0^*) is replaced by Nb^4+^ (4*d*^1^). For example, at 523 K, ρ decreases from 1.64 Ω·cm for *x* = 0 to 0.18 Ω·cm for *x* = 0.10. At 300 K, for *x* = 0, the measured carrier concentration of 1.24 × 10^18^ cm^−3^ is in good agreement with previous measurement on single crystals, and, for *x* = 0.10, it moderately increases up to 2.42 × 10^18^ cm^−3^ confirming that charge carriers are created by the Nb for Ti substitution. Consistently, the absolute value of the Seebeck coefficient decreases with increasing Nb content: At 523 K, *S* = −625 µV/K for *x* = 0 against *S* = −340 µV/K for *x* = 0.10. However, the magnitude of the carrier concentration in the full series remains two orders of magnitude smaller than those obtained in TiS_2_ doped compounds, where values above 10^20^ cm^−3^ are classically measured in undoped compounds. This explains why ρ remains larger in TiS_3_ samples.

**Figure 5:**
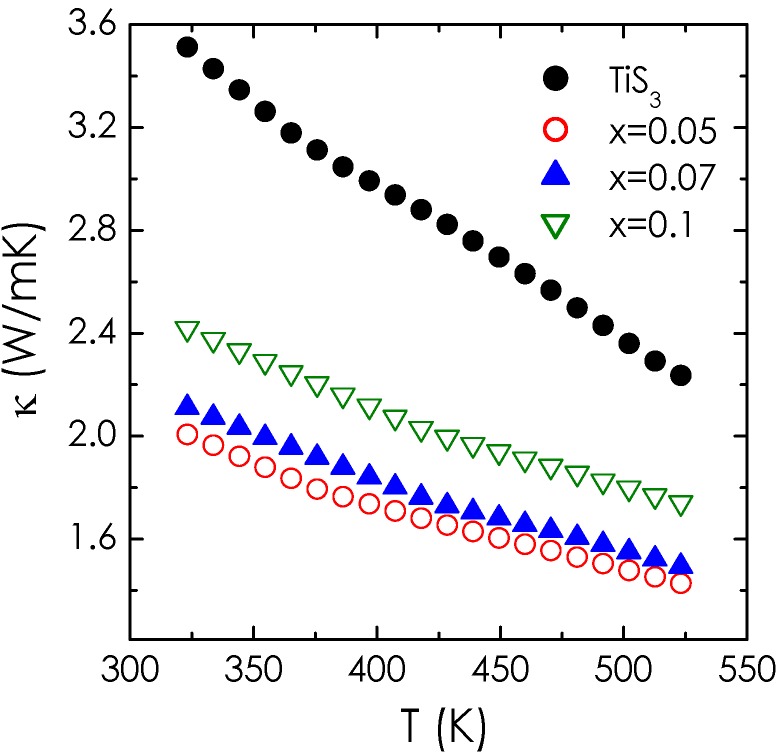
Temperature dependence of the thermal conductivity in the Ti_1 − *x*_Nb*_x_*S_3_ series.

The temperature dependence of the thermal conductivity (κ) in the Ti_1 − *x*_Nb*_x_*S_3_ series is shown in Figure 5. Overall the thermal conductivity of all the substituted compounds decreases with increasing temperature. Nb-substituted compounds show reduced values of the thermal conductivity, below 2.4 W/mK at *RT*, against 3.5 W/mK for TiS_3_. The electronic part of the thermal conductivity being negligible due to the large electrical resistivity, such behavior is mainly governed by the decrease of the lattice thermal conductivity. As already observed before in Ti_1 − *x*_Nb*_x_*S_2_, the decrease of the thermal conductivity with the *x* increase might be explained by mass fluctuations effect due to mixed occupancy of Ti and Nb in the solid solution. However, the coexistence of both variants revealed by the TEM observations could also favor a structural disorder in the stacking directions of the atomic layers also responsible for a κ decrease. Though κ decreases with *x* from *x* = 0.00 to *x* = 0.05 (or *x* = 0.07), the κ increase observed for *x* = 0.10 compared to *x* = 0.05 (or *x* = 0.07) suggests that the electronic part increases, as expected from the ρ decrease. Combining the results for electrical data and thermal conductivity yields the dimensionless figures of merit *ZT* which increases by a factor of 4 as compared to the pristine compound, up to 0.02 at 523 K for *x* = 0.1.

## 3. Experimental Section

The starting precursors used for the synthesis of the title phases were the following elements: titanium, niobium and sulfur (all powders, 99.95%–99.99%, Alfa Aesar). The powders were firstly weighed in the corresponding stoichiometric ratio (total mass of each sample 6 g) according to the formula Ti_1 − *x*_Nb*_x_*S_3_ (*x* = 0, 0.05, 0.07, 0.10, 0.15, 0.20). The mixed powders were placed in a quartz glass tube and sealed under vacuum. The tube was then put into a muffle furnace for reaction. The temperature of the mixture was raised from room temperature to 550 °C with a heating rate of *ca.* 90 °C/h, held for 12 h and then cooled to room temperature with a cooling rate of *ca.* 90 °C/h. The obtained dark gray products were ground using a mortar and sieved down to 200 µm. In order to produce dense samples, Spark Plasma Sintering (SPS) (FCT HPD 25) technique was used. About 4 g of each sample were placed in graphite dies with an inner diameter of 15 mm. The temperature was raised from room temperature to 560 °C with a heating rate of *ca.* 110 °C/min. It was kept at this temperature for 20 min under a pressure of 85 MPa. Finally the samples were cooled down to room temperature with the same rate (*ca.* 110 °C/min). The thickness and diameter of the obtained pellets are around 7 mm and 15 mm, respectively (geometrical density ≥ 95% of the theoretical one in all cases).

Structural characterization was carried out by X-ray diffraction (XRD) analysis using a D8 Advance Vario1 Bruker diffractometer (Cu K*_α_*_1_ radiation, Ge-monochromator, and image plate detector). Electron Dispersive Spectrocopy (EDS) analyses, using an EDAX (Mahwah, NJ, USA) detector on a Zeiss Supra 55 scanning electron microscope, were performed to confirm the cationic compositions.

High angular annular dark field scanning transmission electron microscopy (HAADF-STEM) images and ED patterns was obtained by using JEM ARM200F (JEOL Ltd, Tokyo, Japan) cold field emission gun (FEG) probe and image aberration corrected electron microscope operating at 200 kV and equipped with a large solid-angle CENTURIO Energy Dispersive X-ray spectroscopy (EDX) detector and Quantum Electron Energy Loss Spectroscopy (EELS) spectrometer. Samples for TEM were ground under methanol and transferred to a holey carbon film deposited on Cu supported grid.

Temperature-dependent electrical resistivity (ρ) and Seebeck coefficient (*S*) data were measured simultaneously from 323 to 523 K under partial helium pressure using a ULVAC-ZEM3 (ULVAC-RIKO, Kanagawa, Japan)device. Hall Effect experiments at 300 K have also been carried out in a Physical Properties Measurements Systems (PPMS, Quantum Design), in a magnetic field up to 7 T. Thermal conductivity (κ) was obtained from the product of the heat capacity (calculated using the Dulong–Petit approximation), sample geometrical density and thermal diffusivity (Netzsch LFA457, Netzsch, Selb, Germany) measured under inert atmosphere from 323 to 523 K. All the property measurements were performed on the same puck perpendicular to the SPS pressure direction.

## 4. Conclusions

A new series of compounds Ti_1 − *x*_Nb*_x_*S_3_ (*x* ≤ 0.1) has been successfully synthesized from the elements and structurally characterized. Electron doping by the Nb substitution for Ti induces a substantial decrease in the electrical resistivity and Seebeck coefficient. The formation of the solid solution Ti_1 − *x*_Nb*_x_*S_3_, even in a narrow range of *x* value, is also proved to effectively reduce the lattice thermal conductivity. Due to the low carrier concentration and the semiconductor character of the doped compounds, the too low power factor values leads to *ZT* values that remain smaller by a factor of 50 than those of the TiS_2_ layered compound. To improve the thermoelectric performance, the influence of other transition metals and other chalcogenides when substituting titanium and sulfur in the Ti_1 − *x*_*M_x_*S_3 − *y*_*T_y_* (*M* = Zr, Ta, Hf, W, Rh, and *T* = Se, Te) should be investigated. Furthermore, it would also be necessary to investigate up to which extent titanium can be substituted by *M* in order to increase the carrier concentration in these systems.

## References

[1] Rowe D.M. (2005). Handbook of Thermoelectrics: Macro to Nano.

[2] Imai H., Shimakawa Y., Kubo Y. (2001). Large thermoelectric power factor in TiS_2_ crystal with nearly stoichiometric composition. Phys. Rev. B.

[3] Wan C., Wang Y., Wang N., Koumoto K. (2010). Low-thermal-conductivity (*M*S)_1 + *x*_(TiS_2_)_2_ (*M* = Pb, Bi, Sn) misfit layer compounds for bulk thermoelectric materials. Materials.

[4] Guilmeau E., Bréard Y., Maignan A. (2011). Transport and thermoelectric properties in Copper intercalated TiS_2_ chalcogenide. Appl. Phys. Lett..

[5] Koumoto K., Funahashi R., Guilmeau E., Miyazaki Y., Weidenkaff A., Wang Y.F., Wan C.L. (2013). Thermoelectric ceramics for energy harvesting. J. Am. Ceram. Soc..

[6] Hébert S., Kobayashi W., Muguerra H., Bréard Y., Raghavendra N., Gascoin F., Guilmeau E., Maignan A. (2013). From oxides to selenides and sulfides: The richness of the CdI_2_ type crystallographic structure for thermoelectric properties. Phys. Status Solidi A. Appl. Mater. Sci..

[7] Guilmeau E., Maignan A., Wan C., Koumoto K. (2015). Recent advances in TiS_2_ based compounds: On the effect of substitution, intercalation, non-stoichiometry and block layer concept on electron doping and phonon scattering. Phys. Chem. Chem. Phys..

[8] Beaumale M., Barbier T., Bréard Y., Guelou G., Powell A.V., Vaqueiro P., Guilmeau E. (2014). Electron doping and phonon scattering in Ti_1+*x*_S_2_ thermoelectric compounds. Acta Mater..

[9] Guilmeau E., Berthebaud D., Misse P.R.N., Hébert S., Lebedev O.I., Chateigner D., Martin C., Maignan A. (2014). ZrSe_3_-Type variant of TiS_3_: Structure and thermoelectric properties. Chem. Mater..

[10] Brattas L., Kjekshus A. (1972). On the properties of coumpounds with the ZrSe_3_ type structure. Acta Chem. Scand..

[11] Furuseth S., Brattas L., Kjekshus A. (1975). On the crystal structures of TiS_3_, ZrS_3_, ZrSe_3_, ZrTe_3_, HfS_3_, and HfSe_3_. Acta Chem. Scand. A.

[12] Beaumale M., Barbier T., Bréard Y., Hébert S., Kinemuchi Y., Guilmeau E. (2014). Thermoelectric properties in the series Ti_1 − *x*_Ta*_x_*S_2_. J. Appl. Phys..

[13] Beaumale M., Barbier T., Bréard Y., Raveau B., Kinemuchi Y., Funahashi R., Guilmeau E. (2014). Mass fluctuation effect in Ti_1 − *x*_Nb*_x_*S_2_ bulk compounds. J. Elec. Mater..

[14] Ioffe A.F., Airepetyants S.V., Ioffe A.V., Kolomoets N.V., Stil’bans L.S. (1956). About increasing efficiency of semiconductor thermocouples. Dokl. Akad. Nauk. SSSR.

[15] Zaikina J.V., Kovnir K.A., Sobolev A.N., Presniakov I.A., Kytin V.G., Kulbachinskii V.A., Olenev A.V., Lebedev O.I., van Tendeloo G., Dikarev E.V. (2008). Highly disordered crystal structure and thermoelectric properties of Sn_3_P_4_. Chem. Mater.

[16] Finkman E., Fisher B. (1984). Electrical transport measurements in TiS_3_. Solid State Comm..

[17] Hsieh P.L., Jackson C.M., Grüner G. (1983). Disorder effects in the linear chain compound TiS_3_. Solid State Comm..

[18] Grimmeiss H.G., Rabenau A., Hahn H., Ness P. (1961). Electrical and optical properties of some chalcogenides of elements. Z. Elektrochem..

[19] Grigoryan L.A., Novoselova A.V. (1962). Investigation of niobium sulfides. Dokl. Akad. Nauk SSSR.

[20] Kadijk F., Jellinek F. (1969). The system niobium-sulfur. J. Less Common Metals.

[21] Endo K., Ihara H., Watanabe K., Gonda S. (1981). XPS study on valence band structures of transition-metal trisulfides, TiS_3_, NbS_3_, and TaS_3_. J. Solid State Chem..

[22] Endo K., Ihara H., Watanabe K., Gonda S. (1982). XPS study of one-dimensional compounds: TiS_3_. J. Solid State Chem..

[23] Haraldsen H., Kjekshus A., Rost E., Steffensen A. (1963). On the properties of TiS_3_, ZrS_3_ and HfS_3_. Acta Chem. Scand..

[24] Thompson A.H., Pisharody K.R., Koehler R.F. (1972). Experimental Study of the Solid Solutions Ti*_x_*Ta_1− *x*_S_2_. Phys. Rev. Lett..

[25] Shimakawa M., Maki H., Nishihara H., Hayashi K. (1997). Phase relations and some electrical properties of compounds in TiS2-NbS2 system. Mater. Res. Bull..

